# Quantifying variances in comparative RNA secondary structure prediction

**DOI:** 10.1186/1471-2105-14-149

**Published:** 2013-05-01

**Authors:** James WJ Anderson, Ádám Novák, Zsuzsanna Sükösd, Michael Golden, Preeti Arunapuram, Ingolfur Edvardsson, Jotun Hein

**Affiliations:** 1 Department of Statistics, South Parks Road, Oxford, UK; 2 Bioinformatics Research Center, Aarhus University, Aarhus, Denmark; 3 Computational Biology Group, University of Cape Town, Rondebosch, South Africa; 4 Department of Computer Science, University of North Carolina at Chapel Hill, Chapel Hill, NC, USA; 5 Department of Mathematics, Reykjavik University, Reykjavik, Iceland

## Abstract

**Background:**

With the advancement of next-generation sequencing and transcriptomics technologies, regulatory effects involving RNA, in particular RNA structural changes are being detected. These results often rely on RNA secondary structure predictions. However, current approaches to RNA secondary structure modelling produce predictions with a high variance in predictive accuracy, and we have little quantifiable knowledge about the reasons for these variances.

**Results:**

In this paper we explore a number of factors which can contribute to poor RNA secondary structure prediction quality. We establish a quantified relationship between alignment quality and loss of accuracy. Furthermore, we define two new measures to quantify uncertainty in alignment-based structure predictions. One of the measures improves on the “reliability score” reported by PPfold, and considers alignment uncertainty as well as base-pair probabilities. The other measure considers the information entropy for SCFGs over a space of input alignments.

**Conclusions:**

Our predictive accuracy improves on the PPfold reliability score. We can successfully characterize many of the underlying reasons for and variances in poor prediction. However, there is still variability unaccounted for, which we therefore suggest comes from the RNA secondary structure predictive model itself.

## Background

RNA secondary structure prediction is still an important problem in computational biology. With the advent of next generation sequencing and RNA-seq technologies, many RNA structural changes are being found to play important roles in regulating gene expression [1,2]. Gene regulation studies can now be done on a genome-wide scale. In some cases RNA secondary structures can be experimentally determined on a genome-wide level [3], but these methods require RNA isolation and many not preserve *in vivo* structures. RNA secondary structure prediction programs are still often used to predict structures across the genome [4]. The predicted secondary structures, and predicted structural changes, are being used to find relationships and suggest mechanisms in gene regulatory networks.

Some methods for RNA secondary structure prediction only consider a single sequence as input. However, prediction quality can be improved by using multiple sequences, assuming that RNA secondary structure is conserved through evolution. Even without a complex evolutionary model, these additional structural constraints provide valuable information on folding. Comparative methods for RNA secondary structure prediction are based on this observation, and use evolutionary information from multiple alignments to improve prediction quality.

Methods for RNA secondary structure prediction generally fall into two categories. Thermodynamic models make use of free-energy functions, which take experimentally determined energy parameters for individual structural elements. Dynamic programming is then used to find the secondary structure with the minimum free energy, which is reported as the predicted structure. This has been successfully implemented in programs such as RNAfold [5] and UNAfold [6]. Thermodynamic methods typically deal with the single-sequence prediction problem, but extensions such as RNAalifold [7] and PETfold [8] allow for comparative prediction.

Stochastic context-free grammars (SCFGs), on the other hand, define a probability distribution over the space of RNA secondary structures. Posterior decoding techniques are typically used to determine, for example, the maximum expected accuracy structure [9]. SCFGs have been used for the single-sequence prediction problem [10], but their advantage comes through coupling with a molecular evolution model. The first comparative SCFG-based approach was developed in Pfold [11,12], where alignment column probabilities were determined through single and paired column evolution models, calculated via the Felsenstein pruning algorithm [13]. For a more complete review on RNA secondary structure prediction, see [14].

**Figure 1:**
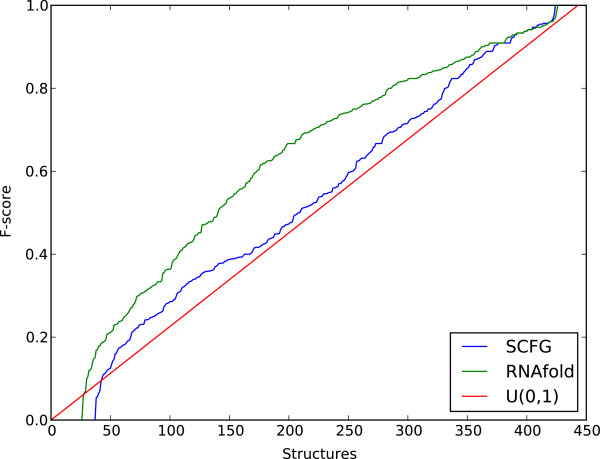
**Cumulative density of secondary structure prediction accuracy on 433 RNASTRAND structures.** Performance accuracy on 433 sequences from RNASTRAND with known secondary structure by RNAfold and PPfold.

In genome-wide predictions of RNA secondary structure, the accuracy of the secondary structure pre-diction program is rarely factored into analysis. Typically only the mean accuracy is reported in RNA secondary structure prediction benchmarks [14], with the variance in the accuracy given little thought. Variance in accuracy is particularly problematic in the case of single-sequence prediction. Figure 1 shows the cumulative density function of predictive accuracy for two single-sequence applications of RNA secondary structure prediction, RNAfold and PPfold (a recent implementation of Pfold, [15]), on 443 sequences taken from the RNASTRAND database [16]. Additionally, a uniform (0,1) cumulative density function is shown for comparison. The figure illustrates that for sequences in this data set, the predictive accuracy of RNAfold and PPfold is not very much different from a random number between 0 and 1. When genome-wide RNA secondary structure prediction is done on a large number of single sequences, many predictions will be poor ones.

Consequently, it is important to understand more about the variability of RNA secondary structure prediction programs. In comparative prediction, there are many sources of variability: alignment quality, the number of sequences chosen and which alignment samples have been taken, the evolutionary relationships between sequences, as well as the ill-conditioned nature of the folding model itself. Understanding and quantifying these variances is key for biological applications that rely on these folding programs. Additionally, other bioinformatics software that utilize these folding programs– for example inverse RNA folding algorithms [17]– may often experience a fundamental limitation in performance due to variance in structure prediction quality.

Sequence alignment is a fundamental step in most comparative sequence analysis pipelines. The typical approach is to create a single, trusted multiple alignment of the sequences using methods based on an artificial scoring scheme and heuristics to find a highly scoring alignment [18,19]. Although this methodology is successful when the alignment is well resolved, it has been shown in the context of downstream analyses that the end result can be highly sensitive to the choice of alignment [20-22]. RNA secondary structure prediction methods take a variety of approaches with respect to possible errors in RNA alignments. Some methods (e.g. [23]) invoke a fold-and-align approach directly, where alignment is done simultaneously with structure prediction. Pfold, instead, takes a fixed alignment as input, but allocates a nite probability to a nucleotide being any other nucleotide; this makes the model more robust to poor alignment quality. Most modern methods (e.g. [24]) still consider prediction from a single, fixed, alignment. Recently, alignment-free methods have also been proposed [25]. However, even after considering poor alignment quality, there are many additional variances associated with poor comparative RNA secondary structure prediction.

Sequence selection is another important variable. Alignment methods may produce poor alignments due to poor individual sequences, which will in turn produce poor structure predictions. There have been methods developed to select homologous sequences, particularly [26], which is based on evolutionary models and structural constraints. This is implemented in [27], which shows strong results in RNA secondary structure prediction can be found by selecting useful sequences.

In this paper we consider the problem of variances in comparative RNA secondary structure prediction. We present statistical analyses of different variances including the relationship between structure prediction quality and chosen alignment distance from the reference alignment, and a predictive algorithm for accuracy is provided. Factors like the number of sequences in the alignment and the evolutionary distance of the sequences are considered. Finally, a novel method is presented which extends information entropy for stochastic context-free grammars [28] to consider variation over alignments.

### Statistical alignment

Statistical alignment [29] provides a solution to many of the issues encountered with the traditional approach of sequence alignment. It models sequence evolution as a stochastic process involving sequence insertions, deletions and character substitutions, which defines a probability distribution over the alignments of the sequences. Using techniques such as Expectation Maximisation or Markov Chain Monte Carlo (MCMC), it is possible to either maximize the likelihood of the alignment, or generate a representative set of putative alignments by sampling from the alignment distribution.

Several statistical alignment implementations have emerged in past years [30-33], some of which allow co-sampling other entities such as the evolutionary tree or the locations of cis-regulatory motifs. Such methods can highlight homoplasy and alignment uncertainty with high accuracy or can be used to decrease alignment uncertainty effects in downstream analyses, for instance protein secondary structure prediction [34].

StatAlign is a statistical alignment software package [32] that allows joint Bayesian analysis of multiple alignments, phylogenetic trees and evolutionary parameters. The background model for insertions and deletions is a modified version of the TKF92 model [35] as described in [34]. The indel model can be coupled with an arbitrary substitution model (many of which are distributed with the software, both for protein and nucleotide sequence data). The Bayesian analysis is based on MCMC, the transition kernels are improved versions of those in [34]. StatAlign generates random samples from the joint posterior distribution of sequence alignments, evolutionary trees and model parameters. This high-dimensional joint distribution can be analysed in several ways, ranging from the simple statistics of marginalised single dimensions (e.g. the posterior distribution of a single rate parameter) to the application of other tools to the alignment samples.

### Alignment and RNA secondary structure accuracy metrics

To analyse variances of RNA secondary structure as alignment quality varies, we calculate a similarity score that measures how close a sample alignment is to the reference alignment. We use an alignment metric, taken from [36], which is generalised to an alignment method in [37].

Let $a_{s_{1}}{,}_{s_{2}}$ be an alignment of a sequence *s*_1_ of length *n* to a sequence *s*_2_ of length *m*. Each column of $a_{s_{1}}{,}_{s_{2}}$ can be expressed as pairs of the form $\left(s_{1}^{i},s_{2}^{j}\right)$, $\left(s_{1}^{i},-\right)$, and $\left(-,s_{2}^{i}\right)$. We define

$$\begin{array}{lll} H\left(a_{s_{1},s_{2}}\right) & = & \left\{\left(i,j\right)|\left(s_{1}^{i},s_{2}^{j}\right)\in a_{s_{1},s_{2}}\right\},\\ I\left(a_{s_{1},s_{2}}\right) & = & \left\{j|\left(-,s_{2}^{j}\right)\in a_{s_{1},s_{2}}\right\},\;\text{and}\\ D\left(a_{s_{1},s_{2}}\right) & = & \left\{i|\left(s_{1}^{i},-\right)\in a_{s_{1},s_{2}}\right\}, \end{array}$$

sets which represent ‘homology’, ‘insertion’ and ‘deletion’ respectively. Given these sets, we define the distance between two alignments $a_{s_{1},s_{2}}^{k}$ and $a_{s_{1},s_{2}}^{\ell }$ of two sequences s_1_ and s_2_ to be

(1) $$\begin{array}{ll} d\left(a_{s_{1},s_{2}}^{k},a_{s_{1},s_{2}}^{\ell }\right) & =n+m-2\left|H\left(a_{s_{1},s_{2}}^{k}\right)\cap H\left(a_{s_{1},s_{2}}^{\ell }\right)\right|\\ & -\left|D\left(a_{s_{1},s_{2}}^{k}\right)\cap D\left(a_{s_{1},s_{2}}^{\ell }\right)\right|-\left|I\left(a_{s_{1},s_{2}}^{k}\right)\cap I\left(a_{s_{1},s_{2}}^{\ell }\right)\right|. \end{array}$$

For example, consider the case $a_{s_{1},s_{2}}^{k}=a_{s_{1},s_{2}}^{\ell }.$ Then we have $n=m,\left|H\left(a_{s_{1},s_{2}}^{k}\right)\cap H\left(a_{s_{1},s_{2}}^{\ell }\right)\right|=n,\left|D\left(a_{s_{1},s_{2}}^{k}\right)\cap D\left(a_{s_{1},s_{2}}^{\ell }\right)\right|=0$ as there are no deletions, and $\left|I\left(a_{s_{1},s_{2}}^{k}\right)\cap I\left(a_{s_{1},s_{2}}^{\ell }\right)\right|=0$ as there are no insertions. This gives the distance between the alignments as zero, as would be expected.

Equation 1 can be generalised to sequence alignments with more than two sequences. Assuming now that *a*^*k*^ and $a^{\ell }$ are alignments of *m* sequences *s*_1_, …, *s*_*m*_ of lengths *n*_1_, …, *n*_*m*_, we have

(2) $$d\left(a^{k},a^{\ell }\right)=\sum_{p=1}^{m-1}\sum_{q=p+1}^{m}d\left(a_{s_{p},s_{q}}^{k},a_{s_{p},s_{q}}^{\ell }\right),$$

that is, summing all the pairwise alignment distances from Equation 1. This alignment metric if is then normalized and subtracted from 1 to produce a similarity score

(3) $$\mathrm{SS}\left(a^{k},a^{\ell }\right)=1-\frac{d\left(a^{k},a^{\ell }\right)}{\left(m-1\right)\sum_{t=1}^{m}n_{t}}$$

The denominator of the fraction, $\left(m-1\right)\sum_{t=1}^{m}n_{t}$, is the normalizing constant, the maximum that the alignment distance $d\left(a^{k},a^{\ell }\right)$ can be. The similarity score is bounded by 0 and 1, with 1 indicating that the sample alignment is identical to the reference alignment.

#### RNA secondary structure metrics

There are a wealth of available metrics on RNA secondary structure [14,38]. Here we use sensitivity, positive predictive value (PPV), and F-score (the harmonic mean of sensitivity and PPV). Defining true positives (TP) as the number of base-pairs correctly predicted, false positives (FP) as the number of true base-pairs not predicted, and false negatives (FN) as the number of base-pairs predicted which are incorrect, we have

$$\begin{array}{lll} \mathrm{Sensitivity} & = & \frac{\mathrm{TP}}{\mathrm{TP}+\mathrm{FN}}\\ \;\mathrm{PPV} & = & \frac{\mathrm{TP}}{\mathrm{TP}+\mathrm{FP}}\\ F-\mathrm{score} & = & \frac{2\times \mathrm{TP}}{2\times \mathrm{TP}+\mathrm{FN}+\mathrm{FP}} \end{array}$$

The strength of these RNA secondary structure accuracy metrics is that they are easy to interpret, and make it straightforward to compare methods across different datasets. An F-score of 1 would represent a structure prediction that was completely correct and an F-score of 0 a structure prediction that only predicted incorrect base-pairs.

### Information entropy

As we later develop calculations for information entropy for a set of alignments, here we outline the computation of information entropy for a single alignment. The information entropy *H* of a probability distribution P with a set of events X is defined as:

(4) $$H\left(P\right)=-\sum_{x\in X}P\left[x\right]\log_{2}\left(P\left[x\right]\right).$$

Information entropy is a measure for the “spread” of the probability distribution, and has well-defined lower and upper bounds. The minimum entropy of 0 occurs when there is only one outcome with probability 1, and the maximum entropy of log_2_ (*n*) occurs when there are *n* possible outcomes, each with probability 1/*n*, that is the uniform distribution. When the base of the logarithm is 2, the entropy is measured in bits. For a probability distribution, an entropy of *k* bits indicates that the expected value of the information content of observing a single outcome is *k* bits. In the context of secondary structure prediction, a low entropy therefore indicates that few secondary structures dominate the probability space, whereas a high entropy indicates a more even probability distribution over possible secondary structures. Thus, information entropy is a useful single quantity to characterize the underlying probability distribution of secondary structures.

The information entropy of the probability distribution over the possible secondary structures generated by a phylo-SCFG can be obtained using expected rule frequencies, which can be computed using the inside-outside algorithm [28]. This is outlined here.

Let the set of all derivations for the input alignment be Φ. Since the probability of a derivation *d* can be written as the product of the SCFG production rule probabilities and the phylogenetic probabilities, we can write the total probability *T* of the grammar producing the input string as

(5) $$T=\sum_{d\in \Phi }P\left[d\right]=\sum_{d\in \Phi }P_{G}\left[d\right]P_{T}\left[d\right],$$

where P_*G*_[*d*] denotes the prior probabilities obtained from the SCFG part of the model, and P_*T*_ [*d*] are the likelihood factors obtained from the phylogenetic model. Conditioning on producing the input string, the normalized probability of a derivation *d* is $P_{\Phi }\left[d\right]=\frac{1}{T}P\left[d\right]=\frac{1}{T}P_{G}\left[d\right]P_{T}\left[d\right]$. Consequently, we have that the information entropy of the input alignment under the phylo-SCFG model is

(6) $$H_{\Phi }\left(G\right)=-\sum_{d\in \Phi }P_{\Phi }\left[d\right]\log_{2}\left(P_{\Phi }\left[d\right]\right),$$

which can be written using Equation 5 as

(7) $$H_{\Phi }\left(G\right)=\log_{2}\left(T\right)-\frac{1}{T}\sum_{d\in \Phi }P\left[d\right]\log_{2}\left(P_{G}\left[d\right]\right)-\frac{1}{T}\sum_{d\in \Phi }P\left[d\right]\log_{2}\left(P_{T}\left[d\right]\right),$$

that is, separating out the SCFG contribution and the phylogenetic contribution. To calculate the entropy in practice, firstly we use a simplified form of the SCFG contribution. For a SCFG with set of production rules *R*, we can write the SCFG contribution in terms of the expected production rule frequency,

(8) $$\sum_{d\in \Phi }P\left[d\right]\log_{2}\left(P_{G}\left[d\right]\right)=\sum_{r\in R}\log_{2}\left(P_{G}\left[r\right]\right)E\left[\mathrm{uses}\;\mathrm{of}\;r\right].$$

Secondly, we can simplify the phylogenetic contribution. Let *r*_*a*_ ∈ *R* be a SCFG rule which produces base pairs, and *r*_*b*_ ∈ *R* a SCFG rule which produces unpaired bases. Define 1^*d*^ (*i*, *j*), the indicator function for whether the column pair (*i*, *j*) is emitted from a rule *r*_*a*_ (i.e. position *i* and *j* form a pair), and 1^*s*^ (*i*), the indicator function for whether column *i* is emitted from a rule *r*_*b*_ (i.e. position *i* is unpaired). Finally, define *f*_*d*_ (*r*_*a*_) as the frequency that rule *r*_*a*_ is used in derivation *d*. Then

$$\begin{array}{ll} \sum_{d\in \Phi }P\left[d\right]\log_{2}\left(P_{T}\left[d\right]\right) & =\sum_{d\in \Phi }P\left[d\right]\left(\sum_{r_{a}},\log_{2},\left(P_{T}{\left[c|c\;\mathrm{unpaired}\right]}^{f_{d}\left(r_{a}\right)}\right)\right)\\ & \;\left(+\sum_{r_{b}}\log_{2}\left(P_{T}{\left[c,c^{\prime }|c,c^{\prime }\right]}^{f_{d}\left(r_{b}\right)}\right)\right)\\ & =\sum_{i}\log_{2}\left(P_{T}\left[i|i\;\mathrm{unpaired}\right]\right)\sum_{d\in \Phi }1^{s}\left(i\right)P\left[d\right]\\ & \;+\sum_{i,j}\log_{2}\left(P_{T}\left[i,j|i,j\;\mathrm{paired}\right]\right)\sum_{d\in \Phi }1^{d}\left(i,j\right)P\left[d\right] \end{array}$$

As $\sum_{d\in \Phi }1^{d}\left(i,j\right)P\left[d\right]$ is the total probability under the model that positions *i* and *j* are emitted from a rule *r*_*a*_, and $\sum_{d\in \Phi }1^{s}\left(i\right)P\left[d\right]$ is just the total probability under the model that position *i* is emitted from a rule *r*_*b*_, the quantity $\sum_{d\in \Phi }P\left[d\right]\log_{2}\left(P_{T}\left[d\right]\right)$ can be computed using the expected rule frequencies obtained through the inside-outside algorithm [39].

## Methods

### Data

#### StatAlign Dataset

To test factors relating to alignment quality and secondary structure prediction quality, a large number of alignment samples from trusted reference alignments with known secondary structures are needed. We have created a curated RNA dataset based on the Rfam database [40] for the purposes of evaluating the framework. Alignments of homologous RNA sequences with known consensus secondary structure were extracted from Rfam seed alignments. From these, 50 RNA families with at least 50 sequences were randomly selected (see Additional file 1) in the section “StatAlign Dataset”. From each family, in a pre-filtering step we removed divergent sequences with long indels, as follows. We defined insertion as consecutive non-gap characters of a sequence in the reference alignment which appear in columns where over 80% of the sequences have gaps. Deletions were defined analogously. Columns with fraction of gaps between 20% and 80% were regarded ambiguous and ignored. To over-penalize long indels, we applied the super linear score function *l* × log_2_ (*l* + 1) for indels of length *l*, indels being defined as above. Then, a sequence was removed from a family if its total indel score was beyond 20 *and* the difference between its indel score and the mean indel score in the family was beyond 3.7 times the standard deviation of the indel scores in family, i.e. if the sequence had significantly more and/or longer indels than what is representative of the rest of the family. Then, 50 sequences were selected at random, and further random selection was done to get pairs, triplets etc. up to 15 sequences in alignments. From these samples of known reference alignment, we could produce many different alignment samples using StatAlign [32]. For each RNA alignment, 200 alignment samples were taken, and the reference alignments were also kept to for comparison. We refer to this dataset throughout as the *StatAlign dataset*.

#### Random alignment data

We also wanted to measure the effect of alignment accuracy on secondary structure independently of alignment method. Therefore we created a dataset based on the RNA families of the StatAlign dataset, where alignments were sampled uniformly at various fixed distances from the reference alignment. Using the alignment distance measure in Eq. 2, we created a Metropolis-coupled MCMC framework that runs several parallel MCMC chains to take alignment samples from the target distribution

(9) $$\pi \left(a\right)=\exp\left(-\frac{2\left|d\left(a,a^{r}\right)-d\right|}{t}\right)$$

where *a*^*r*^ is the reference alignment, d is the target distance to get samples from and *t* is the temperature of the chain. To improve the mixing properties of the chains we allowed each chain to explore alignments that do not exactly match the specified target distance (with an exponentially decreasing probability, as described by Eq. 9) but then rejected non-exact matches when taking samples from the cold chain (*t* = 1).

The state space of the Markov chains is the set of all possible multiple alignments of the input sequences. Alignments that represent the same set of homology statements, and only differ by the order of the alignment columns, are treated as different (e.g. alignments $\begin{array}{c} A-\\ -B \end{array}$ and $\begin{array}{c} -A\\ B- \end{array}$ of the sequences A and B). The following basic alignment rearrangement moves are iterated:

1. breaking an alignment column into two columns by moving one of its characters into a new column, placing gaps in every other row:

$$\begin{array}{c} \begin{array}{ccc} \begin{array}{l} C\\ C\\ C \end{array} & \left|\begin{array}{l} A\\ A\\ A \end{array}\right| & \begin{array}{l} G\\ G\\ G \end{array} \end{array}\\ \overset{1.}{\underset{2.}{⇌}}\\ \begin{array}{ccc} \begin{array}{l} C\\ C\\ C \end{array} & \left|\begin{array}{l} -A\\ A-\\ A- \end{array}\right| & \begin{array}{l} G\\ G\\ G \end{array} \end{array} \end{array}$$

2. the exact reverse of the previous, i.e. joining two compatible adjacent columns – one having gaps in all but one row, the other having a gap in that row – to form a single column (see above)

3. relocating one character of a column to a gap position of an adjoining column:

$$\begin{array}{c} \begin{array}{ccc} \begin{array}{l} C\\ C\\ C \end{array} & \left|\begin{array}{l} \mathrm{AG}\\ \mathrm{AG}\\ A- \end{array}\right| & \begin{array}{l} T\\ T\\ T \end{array} \end{array}\\ \overset{3.}{\Leftrightarrow }\\ \begin{array}{ccc} \begin{array}{l} C\\ C\\ C \end{array} & \left|\begin{array}{l} \mathrm{AG}\\ \mathrm{AG}\\ -A \end{array}\right| & \begin{array}{l} T\\ T\\ T \end{array} \end{array} \end{array}$$

As the space of alignments is vast and the moves are very local, to get a good approximation of uniform sampling, the number of steps that have to be done is on the order of millions, even for small inputs when a single chain is run. To this end, we created a special alignment representation where the above rearrangements can be carried out very efficiently (essentially constant time, i.e. in time proportional to column size but independent of the alignment length, even when the cost of randomly selecting the column to alter is included). For moderate input sizes (5–10 sequences of length 300–500) this representation allowed us to take 1.5 million rearrangement steps in a second (using a Java 6 implementation on one core of a 2.4 GHz Intel i3 processor).

A single chain is sufficient for small alignments, but very slow mixing becomes an issue for practical alignment sizes. We have found that standard parallel tempering techniques effectively speed up mixing if the chain temperatures are chosen correctly. Because the optimal temperatures vary significantly depending on reference alignment characteristics and target distribution, a simple acceptance optimization routine was added that tunes chain temperatures to achieve chain swap acceptance ratios between neighboring chains around a pre-set value. We found ratios 0.7–0.8 to be the most effective. With this framework, running 8–10 parallel chains was best to maximize the speed of convergence to the uniform distribution as compared to a single chain with sampling times 8–10-fold.

The above described framework was utilized to create a dataset consisting of the RNA families of StatAlign dataset, where for each family and each selection of 5 representative sequences from the family, 10 samples were taken at a distance corresponding to a similarity of 0.98 to the reference alignment (see Eq. 3), then 10 samples at a similarity of 0.96 etc., down to a similarity ratio of 0.6. We refer to this dataset as the *Random Alignment dataset*.

### Extending information entropy to alignment space

The information entropy defined for a single alignment contains the length of the alignment as a parameter. Attempting to extend the measure to the probability mass over RNA secondary structure space, variable alignment length is a concern. For example, if we have two alignments and corresponding secondary structures

$$\begin{array}{cc} \left(\;(\;(\;\left(\;.\;.\;.\;.\;.\;\right)\;)\;)\;\right) & \left(\;(\;(\;\left(\;....\;\right)\;)\;)\;\right)\\ \mathrm{CCCCAAAA}-\mathrm{GGGG} & \mathrm{CCCCAAAAGGGG}\\ \mathrm{CCCCAAA}-\mathrm{AGGGG} & \mathrm{CCCCAAAAGGGG} \end{array}$$

we would not want to suggest that these alignments give two different secondary structures. Consequently, we use a projection method to give alignment column pairing probability matrices the same dimension, so that the matrices can be averaged.

For a given set of input sequences, the sequence containing the greatest number of non-gap characters was chosen as the reference sequence. Each pairing probability matrix is projected by deleting columns and rows of the matrix corresponding to gap positions in the reference sequence, thus ensuring each matrix corresponding to an alignment sample has the same dimensions (a square matrix, with dimensions equal to the number of non-gap characters in the reference sequence). For example, we might start with an (*n* + 1) (*n* + 1) matrix, delete row *i* and column *i* due to a gap in position *i* in the reference sequence, then end up with an n × n matrix as required.

To calculate information entropy over alignments, we need to be able to calculate the probability of each alignment. However, we cannot calculate the information entropy explicitly, since the probability of a given secondary structure *ss* is

(10) $$P\left[\mathrm{ss}\right]=\sum_{A\in \;\text{alignments}}P\left[\mathrm{ss}|A\right]P\left[A\right],$$

and there is no known efficient way to recurse over all possible alignments. Instead, we create an information entropy measure based on samples from the alignment space, and show that, in the sample-size limit, the alignment-sample information entropy tends to the true information entropy.

Consider alignment samples *a*_1_, …, *a*_*n*_ from the space of all alignments of *m* sequences, sampled according to their probability. If we are using statistical alignment, as in StatAlign, we will be sampling alignments in this fashion. Then we have for a column c, once alignments have been projected to the same length, the probability of being unpaired

(11) $$\begin{array}{l} P\left[c|c\;\mathrm{unpaired}\right]=\sum_{i=1}^{n}P\left[c|c\;\mathrm{unpaired}\;\mathrm{in}\;\mathrm{alignment}\;a_{i}\right]P\left[a_{i}\right], \end{array}$$

with an analogous result holding for paired columns. We now define a sample phylogenetic probability P_*S*_ as the average of the sample phylogenetic probabilities:

(12) $$P_{S}\left[c|c\;\mathrm{unpaired}\right]=\sum_{i=1}^{n}\frac{1}{n}P\left[c|c\;\mathrm{unpaired}\;\mathrm{in}\;\mathrm{alignment}\;a_{i}\right],$$

To show this sample probability converges to the true probability as sample size tends to infinity, we first note that, rearranging Equation 12:

(13) $$\begin{array}{l} P_{S}\left[c|c\;\mathrm{unpaired}\right]\\ =\sum_{i=1}^{n}\frac{1}{n}\left(\sum_{A\in \mathrm{alignments}}P\left[c|c\;\mathrm{unpaired}\;\mathrm{in}\;\mathrm{alignment}\;A\right]1_{\left\{A=a_{i}\right\}}\right), \end{array}$$

with 1 being the indicator function. Hence

(14) $$\begin{array}{l} P_{S}\left[c|c\;\mathrm{unpaired}\right]\\ =\sum_{A\in \mathrm{alignments}}P\left[c|c\;\mathrm{unpaired}\;\mathrm{in}\;\mathrm{alignment}\;A\right]\left(\sum_{i=1}^{n}\frac{1}{n}1_{\left\{A=a_{i}\right\}}\right). \end{array}$$

Taking the limit *n* → *∞*, by the weak law of large numbers

(15) $$P_{S}\left[c|c\;\mathrm{unpaired}\right]\;\to \;\sum_{A\in \;\mathrm{alignments}}P\left[c|c\;\mathrm{unpaired}\;\mathrm{in}\;\mathrm{alignment}\;A\right]P\left[A\right]\;\mathrm{as}\;n\to \infty$$

(16) $$=P\left[c|c\;\mathrm{unpaired}\right]$$

as required.

Now, we have from above that the entropy *H*_Φ_ (P) of grammar derivations Φ of a grammar *G* is

(17) $$\begin{array}{ll} H_{\Phi }\left(P\right)=\log_{2}\left(T\right) & -\frac{1}{T}\sum_{d\in \Phi }P_{G}\left[d\right]P_{T}\left[d\right]\log_{2}\left(P_{G}\left[d\right]\right)\\ & -\frac{1}{T}\sum_{d\in \Phi }P_{G}\left[d\right]P_{T}\left[d\right]\log_{2}\left(P_{T}\left[d\right]\right) \end{array}$$

Since tree probabilities are the product of unpaired and paired column probabilities in the derivation, the tree probabilities can be recalculated from the sample of alignments. These can then be substituted into the above equation to get an approximation to the information entropy over the space of sampled alignments as well. We refer to this entropy of more than one alignment as the *alignment consensus entropy*.

## Results and discussion

### Alignment quality and predictive accuracy

A common question in comparative RNA secondary structure prediction is how many sequences are required to get a good structure prediction. This is briefly addressed in [11], but the sample size is quite small, and only total accuracy is considered. With 15 sequences in the alignment, we assume that no more evolutionary information can be gained by adding further sequences, but when fewer sequences are present, lack of information might yield a poorer structure prediction.

Instead of considering total accuracy, we wanted to quantify relatively how much accuracy is lost when fewer sequences are present. For example, if an alignment with 15 sequences is predicted with an average F-score of 0.5, and an alignment of the same family with 3 sequences is predicted with an average F-score of 0.4, then 80% of the accuracy will have been retained, that is the alignment with 3 sequences has a *relative F-score* of 0.8.

To investigate how many sequences are needed for a good structure prediction, we took the StatAlign dataset and considered the relative F-score for each family. Almost 100% of the possible F-score was achieved when the alignment contained 5 sequences, for both PPfold and RNAalifold. Interestingly, the accuracy of RNAalifold decreased slightly as the number of sequences was increased. This is due to the increased number of non-canonical base-pairs in the alignments, which the thermodynamic method could not predict. PPfold, on the other hand, has a small probability for non-canonical base-pairs, so is not affected by these in the same way. Overall these results suggest that 5 sequences are sufficient for approaching maximal predictive accuracy.

**Figure 2:**
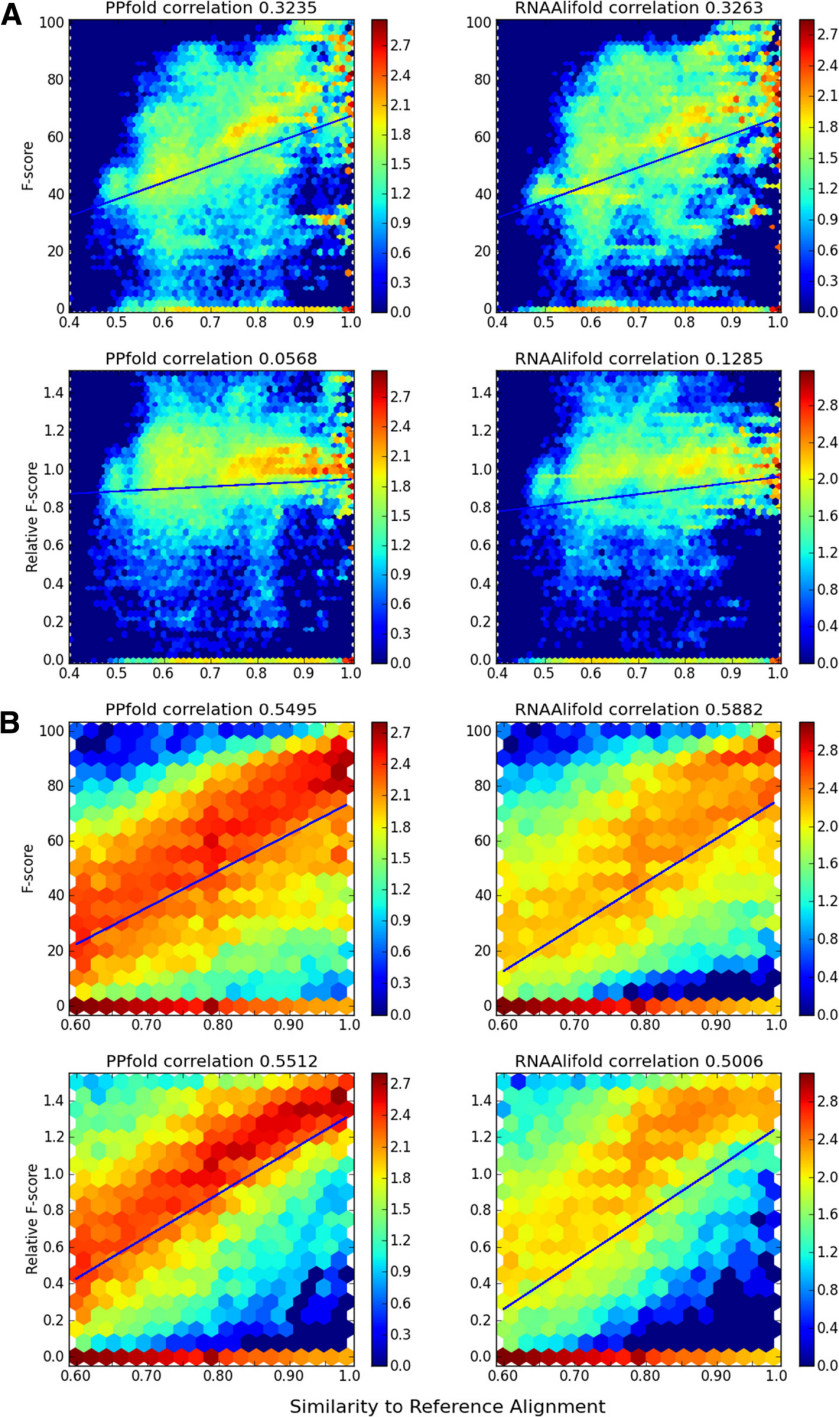
**Log-scale heatmaps for mean accuracy, and for percentage of accuracy retained, when alignment quality varies.** Performance accuracy on the StatAlign dataset **(A)** and Random Alignment dataset **(B)** for PPfold and RNAalifold when alignment quality is varied. Percentage of accuracy is determined by, for each family, normalising by the average accuracy on the reference alignment. The *R*^2^ correlations given are determined by a linear regression model.

To consider the effect alignment quality has on RNA secondary structure prediction, we took the StatAlign and Random Alignment datasets and measured their similarity to the reference alignment using the similarity score above. Again, percentage of accuracy retained was calculated by normalizing against the accuracy achieved on the reference alignment. Log-scale heatmaps showing the accuracy and percentage of accuracy retained for the StatAlign dataset (A) and Random Alignment dataset (B) for PPfold and RNAalifold can be seen in Figure 2. As expected, decreasing alignment quality decreases the accuracy of structure predictions. However, other patterns also emerge from these graphs.

Firstly, in the StatAlign dataset (Figure 2A), we observe a weaker correlation between alignment distance and accuracy than for the Random Alignment dataset (Figure 2B). This suggests that predictions are better for the StatAlign dataset on alignments far from the reference alignment. StatAlign looks to produce alignments with a high likelihood under an evolutionary model, which the random alignments do not consider, and so StatAlign's alignments could be considered more realistic. This con rms the expectation that StatAlign's alignments are more useful for RNA secondary structure prediction than random alignments.

Secondly, for the StatAlign dataset, we see a much higher correlation with the F-score than with the relative F-score. Some families of RNA consistently produce the same alignment, which skews the graphs. For example, an alignment which consistently has similarity to the reference alignment of 0.9, and F-score 0.5 would give a relative F-score of 1 every time, and would support the correlations seen on each graph. Because we can control the spread of distances in the random alignment data set, we don't see this behaviour. As expected, variation comes more with families that produce more variable alignments. In the StatAlign dataset, this is obscured by those families which produce consistent alignments.

Lastly, for the Random Alignment dataset (Figure 2B), we see many more zero quality predictions in the case of RNAalifold than in the case of PPfold. This is most easily seen by the larger intensity of red in the main body of the heatmap for PPfold. This suggests that PPfold functions better than RNAalifold when given a low-quality alignment, likely due to its more complete model for molecular evolution.

#### Evolutionary distance

We also consider the effects of evolutionary distance on RNA secondary structure prediction quality. One might expect that there is a “sweet spot” for evolutionary distance– sequences too close to each other do not display enough co-variation to benefit the evolutionary model, but the evolutionary signal might be lost if the distance is too large. To investigate this, we measured evolutionary distance in the phylogenetic trees predicted by PPfold using four different measures:

**Measure 1–** Mean of all the evolutionary distances,

**Measure 2–** Standard deviation of all the evolutionary distances,

**Measure 3–** Maximum evolutionary distance,

**Measure 4–** Maximum difference between evolutionary distances.

All four measures would be expected to be correlated with sequence length, which is well known to correlate with predictive accuracy. To account for this, we considered relative evolutionary distance, similar to the relative predictive accuracy above. A measure was normalized by the average for that family and number of sequences, so that it could be seen whether an alignment had greater or less evolutionary distance than might be expected. We then looked at the correlation between relative evolutionary distance and predictive accuracy. All methods correlated extremely poorly with predictive accuracy and relative predictive accuracy, measures 1 to 4 having *R*^2^ correlations with relative predictive accuracy of 0.0259, 0.0373, 0.0303, and 0.0265 respectively (data not shown). This suggests that evolutionary distance is not an underlying factor for variation in RNA secondary structure prediction, and those other factors, such as those seen in [26], play a more important role in determining predictive accuracy.

### Alignment distances and maximum posterior decoding

Given the results concerning accuracy lost as alignment quality decreases, it would be desirable to be able to predict alignment quality, with the hope of predicting structure prediction quality. This has previously been attempted in [36]. First, the sequences were aligned with ClustalW [18]. The sequences were then re-aligned using 4 other programs (Align-m [41], MUSCLE [42], Prob-Cons [43], and T-Coffee [44]) and the similarity between the alignment generated using ClustalW to each of the 4 other alignments was measured. The maximum of the 4 similarities, max (*g*), was chosen as a predictor of alignment quality. The authors of [36] detected a strong correlation between the true similarity (the similarity between the ClustalW alignment and a reference alignment) and max (*g*).

We implemented a modified version of this method. For a given set of input sequences we aligned with both AMAP [36] and with StatAlign, obtaining the maximum posterior decoding alignment (MPD alignment) from StatAlign. The similarity between the AMAP alignment and the MPD alignment was used as our predicted similarity measure. This produced a strong correlation between our predicted similarity and true similarity, with an adjusted R-squared value of 0.6524.

**Figure 3:**
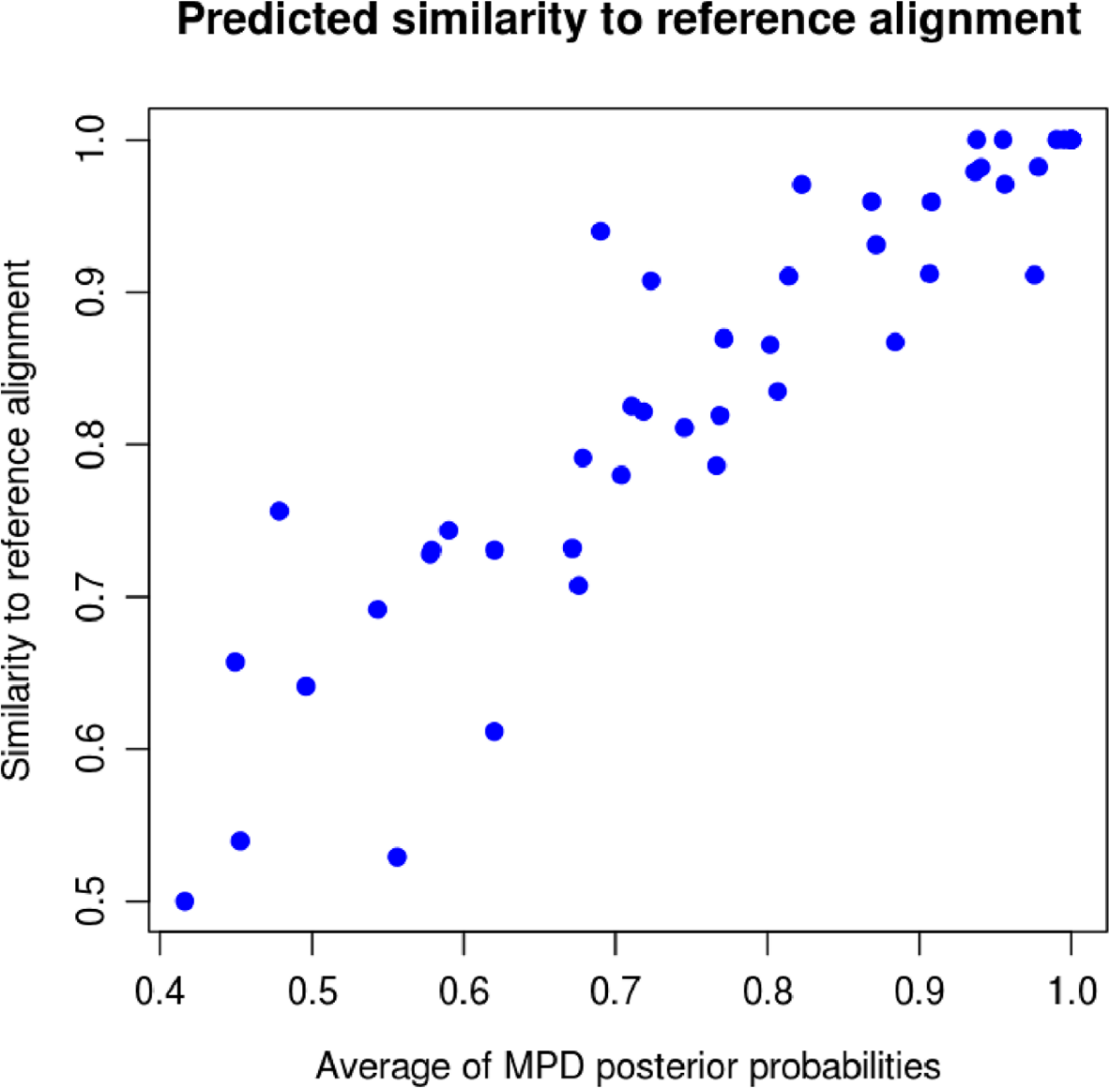
**Predicted similarity with the reference alignment.** Similarity with the reference alignment against predicted similarity with the reference alignment for 50 Rfam families with 5 sequences in. Predicted similarity was calculated by averaging the posterior probabilities given in the MPD alignment. The adjusted R-squared value for a linear fit is 0.8403.

We also implemented another method, which calculates an estimate of the expected similarity score using posterior probabilities from the MPD alignment. For each column, we might expect that a posterior probability close to 1 would contribute a score of close to 1 to the similarity measure. So our predicted alignment distance is just the average of the column posterior probabilities. Figure 3 shows an example of the correlation between predicted similarity and true similarity, here giving an adjusted R-squared value of 0.8403. Our new predicted similarity can be calculated efficiently, and is a strong predictor of true similarity to the reference alignment.

### Extending information entropy to alignment space

**Figure 4:**
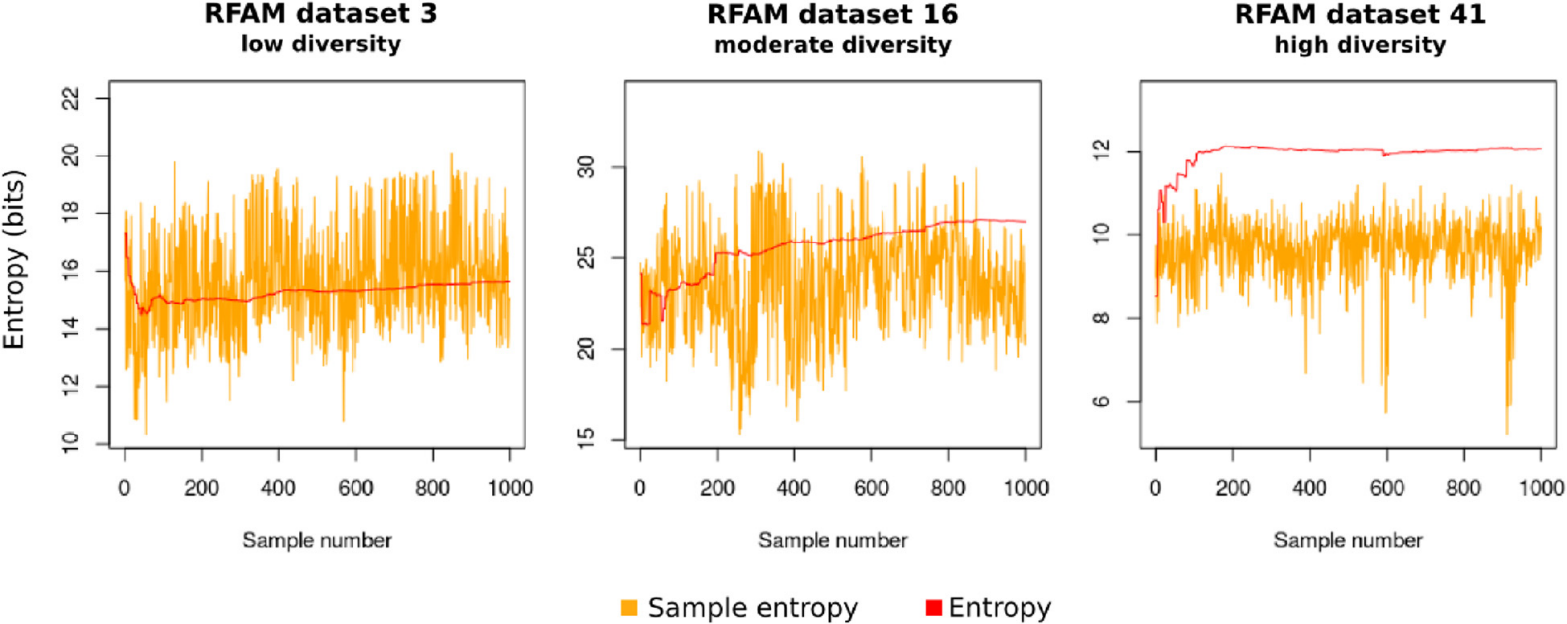
**Information entropy with alignment uncertainty.** Information entropy calculated for each alignment sample (yellow) and the alignment consensus entropy (blue). The leftmost figure is one where the alignment samples were very similar, the rightmost figure where the alignment samples were very different, and the middle figure closer to the median value of alignment sample similarity. See Methods section for details on entropy calculation.

To test the information entropy extension developed above, we calculated the alignment consensus entropy for samples of alignments from the StatAlign dataset. Figure 4 gives information entropy for 3 different representative RNA families from the StatAlign dataset. On each graph, the information entropy for each of 1000 statistical alignment samples is given, as well as the alignment consensus entropy. The leftmost figure is one where the alignment samples were very similar, the rightmost figure where the alignment samples were very different, and the middle figure closer to the median value of alignment sample similarity. For family 3 (where sample alignments had low diversity), we see that the alignment consensus entropy is comparative to the mean entropy of the individual samples. This is expected, as it indicates there is little uncertainty in the alignment. On the other hand, the high-diversity family has much higher alignment consensus entropy than for each individual sample. This is again expected, as the difference in entropies indicates a high uncertainty in alignment. In this way, we can incorporate alignment uncertainty into our understanding of comparative RNA secondary structure prediction.

### Predicting secondary structure accuracy

Given strong correlations between the alignment quality and the structure prediction quality, we might expect that we could find a predictor of structure prediction accuracy. By “integrating out” alignment uncertainty, we may find a reliability score which is more reliable than the one currently reported by the PPfold. To test this, we predicted accuracy for one of the five-sequence alignments of each family and then tested the predicted accuracy against the true accuracy. The PPfold reliability score produced an adjusted *R*^2^ score of 0.252 when considering correlation with the true F-scores.

**Figure 5:**
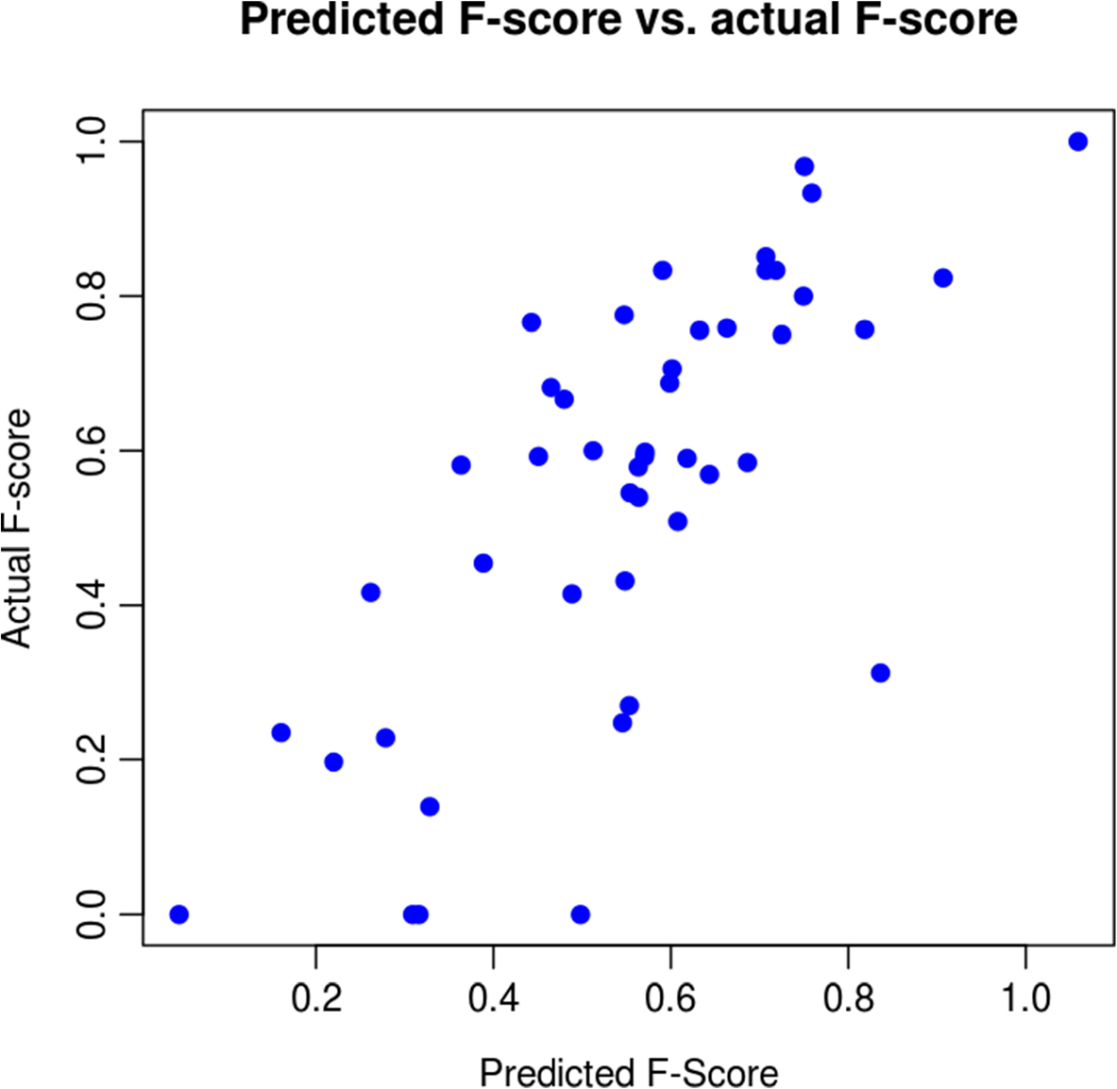
**Predicted RNA secondary structure accuracy.** Predicted F-score of PPfold on MPD alignment against actual F-score of PPfold on MPD alignment. The adjusted R-squared value a linear fit is 0.496.

For our new reliability score, we adjusted the PPfold reliability score to consider only base-pairs, as the F-score considers only base-pairs (i.e. ignored unpaired nucleotide probabilities). We then performed linear regression with the average of the MPD column probabilities, the information entropy of the alignment space, and this pairs-only reliability score against the known F-score measure. This multiple regression improved the reliability score significantly. Figure 5 shows the predicted F-score against the true F-score, for a randomly chosen five-sequence alignments from each family of the StatAlign dataset. The adjusted *R*^2^ value with the new reliability measure improved to 0.496. These results seem to indicate that while alignment quality does affect structure prediction quality, the actual structure prediction model still plays a great role in the overall prediction accuracy. Consequently, improving these models, possibly by incorporating other kinds of information (such as experimental probing data), is an area where new research e orts are still needed in RNA secondary structure prediction.

## Conclusions

In this paper we have explored a number of factors which can contribute to poor RNA secondary structure prediction quality. We established a relationship between alignment quality and expected loss of accuracy. Furthermore, we provided a method to predict alignment quality based only on statistical alignment samples. While our predictor of accuracy improves on the PPfold reliability score, there is still a large amount of variability unaccounted for, which we therefore suggest comes from the predictive model itself. To consider this further, we extended the information entropy measure for SCFGs to consider uncertainty in alignments.

The fact that our accuracy predictor did not account for all the variances associated with RNA secondary structure prediction, despite good predictors being found for alignment quality and a strong correlation between alignment quality and predictive accuracy, suggests that whilst alignment quality is an important factor, the predictive model itself determines plays a large part in the quality of prediction. Given what is shown in Figure 1 for single sequence predictions, that the accuracy of PPfold and RNAfold is very variable, it is unsurprising that variances remain. Clearly then, further efforts should be put into creating stronger single-sequence models, and then the advantages of evolutionary modelling and additional structural constraints will benefit further. The use of experimental data from new probing experiments as well as more biologically realistic constraints, such as kinetic or co-transcriptional folding, may improve the results of RNA secondary structure prediction.

## Competing interests

The authors declare they have no competing interests.

## Authors’ contributions

JWJA, AN, and ZS formulated the ideas presented here, carried out some of the analyses, and wrote the manuscript. AN generated the StatAlign and Random Alignment datasets, and MG, PA, and IE generated some of the results for the reliability scores, alignment distances, and predictive algorithm. JWJA and ZS developed the theoretical framework for extending information entropy. All authors read and approved the final manuscript.

## Supplementary Material

Additional file 1 Information on the 50 RNA families randomly selected from Rfam. Click here for file
